# Serum Metabolic Profiling Analysis of Chronic Gastritis and Gastric Cancer by Untargeted Metabolomics

**DOI:** 10.3389/fonc.2021.636917

**Published:** 2021-03-11

**Authors:** Lin Yu, Qinhuai Lai, Qian Feng, Yuanmeng Li, Jiafu Feng, Bei Xu

**Affiliations:** ^1^Departmant of Clinical Laboratory, Mianyang Central Hospital, School of Medicine, University of Electronic Science and Technology of China, Mianyang, China; ^2^Collaborative Innovation Center for Biotherapy, West China Hospital, West China Medical School, Sichuan University, Chengdu, China; ^3^College of Medical Technology, Chengdu University of Traditional Chinese Medicine, Chengdu, China; ^4^Department of Medical Laboratory, Affiliated Hospital of Southwest Medical University, Luzhou, China

**Keywords:** chronic gastritis, gastric cancer, untargeted metabolomics, lipid metabolism, candidate biomarkers

## Abstract

**Purpose:**

Gastric cancer is a common tumor of the digestive system. Identification of potential molecules associated with gastric cancer progression and validation of potential biomarkers for gastric cancer diagnosis are very important. Thus, the aim of our study was to determine the serum metabolic characteristics of the serum of patients with chronic gastritis (CG) or gastric cancer (GC) and validate candidate biomarkers for disease diagnosis.

**Experimental Design:**

A total of 123 human serum samples from patients with CG or GC were collected for untargeted metabolomic analysis *via* UHPLC-Q-TOF/MS to determine characteristics of the serum. Principal component analysis (PCA), partial least squares discriminant analysis (PLS-DA), and heat map were used for multivariate analysis. In addition, commercial databases were used to identify the pathways of metabolites. Differential metabolites were identified based on a heat map with a *t*-test threshold (*p* < 0.05), fold-change threshold (FC > 1.5 or FC < 2/3) and variable importance in the projection (VIP >1). Then, differential metabolites were analyzed by receiver operating characteristic (ROC) curve to determine candidate biomarkers. All samples were analyzed for fasting lipid profiles.

**Results:**

Analysis of serum metabolomic profiles indicated that most of the altered metabolic pathways in the three groups were associated with lipid metabolism (*p* < 0.05) and lipids and lipid-like molecules were the predominating metabolites within the top 100 differential metabolites (*p* < 0.05, FC > 1.5 or FC < 2/3, and VIP >1). Moreover, differential metabolites, including hexadecasphinganine, linoleamide, and N-Hydroxy arachidonoyl amine had high diagnostic performance according to PLS-DA. In addition, fasting lipid profile analysis showed the serum levels of total cholesterol (TC), high-density lipoprotein cholesterol (HDL-C) and apolipoprotein A1 (Apo-A1) were decreased concomitant to the progression of the progression of the disease compared with those in the control group (*p* < 0.05).

**Conclusions:**

Thus, this study demonstrated that lipid metabolism may influence the development of CG to GC. Hexadecasphinganine, linoleamide, and N-Hydroxy arachidonoyl amine were selected as candidate diagnostic markers for CG and GC.

## Introduction

Gastric cancer (GC) is a common digestive system tumor worldwide with a five-year survival rate ranging from 30 to 65% (1). The development of GC is a complex process involving environmental factors and molecular changes at the cellular level (2). *Helicobacter pylori (H. pylori)* infection is the primary risk factor for gastritis, and chronic gastritis (CG) is the main cause of GC. Great efforts have been made to the diagnose and treat this disease; however, the mortality rate has remained essentially unchanged worldwide in the past decades (3). Currently, clinicians extensively use diagnostic and prognostic biomarkers to improve the clinical course of GC (4, 5). However, gastric carcinoma involves mixed cell types with variable degrees of differentiation, and the majority of serum biomarkers have low sensitivity and low positive predictive value, such as CEA, CA19-9 and CA72-4 (6–8). Therefore, investigation of the mechanisms of the development and identification of novel biomarkers with high sensitivity and specificity for GC is very important for decreasing the incidence and mortality of GC.

Metabolomic technologies, especially untargeted metabolomic approaches, often provide additional information about the global profiling of the metabolome and are used to explore new mechanisms of carcinogenesis (9, 10). Metabolomic technology is also a powerful tool for the discovery of key differential metabolites that can be used as biomarkers in various tumors, such as breast cancer (11), epithelial ovarian cancer (12), lung cancer (13), GC (14). Metabolites of four major classes of biomolecules (carbohydrates, amino acids, lipids, and nucleic acids) were altered in GC according to the results of metabolomic analysis of cell lines, serum, plasma, urine or gastric juice (15–17). However, the results across different studies are inconsistent apparently due to different sensitivity of metabolomic methods (18), variability of experimental subjects, and differences in the number of samples. Additionally, the values of biomarkers should be validated. Furthermore, the investigations of the mechanism of alterations in the metabolism and specific metabolic pathways in GC are relatively insufficient; thus, it is difficult to clearly define metabolic changes in this disease based on metabolomic data. Overall, additional exploration of metabolic disorders for gastric carcinogenesis is needed (16).

Serum markers can reflect systemic metabolic deregulation in patients. Non-invasive and inexpensive serum biomarkers are more suitable for clinical application. Therefore, the serum metabolomic profiles of patients with CG or GC were studied by untargeted metabolomics to identify significantly altered pathways and differential metabolites, and fasting lipid profiles were also investigated. This study may provide insights into the pathogenesis of GC, and the candidate biomarkers may be used to diagnose CG and GC.

## Materials and Methods

### Design, Setting and Participants

A total of 123 subjects were enrolled in the clinical laboratory of Mianyang Central Hospital over a period of 6 months (June to December 2019); the protocol was approved by the Ethics Committee of Mianyang Central Hospital. The control group included 40 patients (20 males and 20 females, age range 27–78 years), the CG group included 32 patients (13 males and 19 females, age range 14–78 years), and the GC group included 51 patients (40 males and 11 females, age range 28–70 years). Inclusion criteria for the healthy control group were: 1) no diseases, such as hypertension, cardiovascular disease, diabetes, and tumor, in the medical history; 2) normal indicators of the functional capacity of several critical organs and systems; 3) no other infections (including *H. pylori* infection) or other diseases that affect gastric function; 4) the test results for all tumor markers performed in our laboratory were within the reference interval within a month before sampling; and 5) no health products or medicines that influence gastric function testing have been used within a month before sampling. Exclusion criteria for the disease groups were: 1) failure to collect blood as required; 2) women in menstruation, pregnancy, or lactation; 3) metabolism-related diseases and other digestive diseases; and 4) some factors, such as diet and lifestyle that influence gastric functions. In addition, all diagnoses were confirmed by a senior clinician according to the clinical diagnostic criteria; the detailed clinical data are shown in **Table 1**. Blood from the control subjects and CG and GC patients was collected after fasting; the tests included fasting lipid profile assays and untargeted metabolomic analysis.

**Table 1:** Clinical characteristics of the subjects.

Group	Control (n = 40)	CG(n = 32)	GC(n = 51)	*χ^2^/F* value	*p* value
Male/female (n)	20/20	13/19	40/11^*^	13.797	0.001
Age (years ± standard deviation)	47.33 ± 15.47	49.91 ± 14.73	54.61 ± 10.33^*^	3.721	0.029
**History**					
*H. pylori* infection	0	7	0	NA	NA
Gastritis (n)	0	32	NA	NA	NA
Chronic active gastritis (n)	0	30	NA	NA	NA
Intestinal metaplasia and/or atrophy (n)	0	2	NA	NA	NA
**Endoscopic diagnosis**					
Normal	NA	2	NA	NA	NA
Hiatal hernia	NA	0	NA	NA	NA
Esophagitis	NA	0	NA	NA	NA
Antroduodenitis	NA	0	NA	NA	NA
Duodenal ulcer	NA	0	NA	NA	NA
Gastric ulcer	NA	10	NA	NA	NA
Other	NA	20	NA	NA	NA
**Tumor localization**					
Antrum	NA	NA	20	NA	NA
Corpus	NA	NA	10	NA	NA
Cardias	NA	NA	8	NA	NA
Unknown	NA	NA	13	NA	NA
**Histologic grade**					
Grade 1	NA	NA	8	NA	NA
Grade 2	NA	NA	9	NA	NA
Grade 3	NA	NA	11	NA	NA
Grade 4	NA	NA	23	NA	NA
**TNM stage**					
I	NA	NA	9	NA	NA
II	NA	NA	3	NA	NA
III	NA	NA	7	NA	NA
IV	NA	NA	11	NA	NA
Unknown	NA	NA	21	NA	NA

Compared with the control group, ^*^p < 0.05.

### Sample Preparation for UHPLC-Q-TOF/MS Analysis

All serum samples were collected into 5 ml tubes (BD Vacutainer^®^ SST II Advance tube) in the morning after fasting for 8–14 h according to the criteria. The samples were centrifuged at 3,000 rpm for 15 min and assayed within 4 h or stored at −80°C until analysis (19). Internal standard (IS) solution (10 µl; 10 μg/ml, clenbuterol for the positive ion mode and chloramphenicol for the negative ion mode analyses) and 800 µl of methanol–acetonitrile (1:1 v/v) were mixed with a 190 µl aliquot of the serum samples; the mixture was vortexed for approximately 30 s and sonicated for 10 min at 4°C in a water bath. Then, the mixture was incubated −20°C for 1 h and centrifuged at 13,000 rpm for 15 min at 4°C. Then, the supernatant (800 µl) was carefully removed, transferred to another clean test tube, and evaporated to dryness under nitrogen at room temperature. The dried residue was reconstituted in 200 µl of 80% methanol and mixed by vortexing for 2 min and sonication for 10 min at 4°C. After centrifuging at 13,000 rpm for 15 min at 4°C, the supernatant was filtered through Acrodisc GHP 0.2 μ, 13 mm Minispikes, and a 5 µl aliquot of the filtrate was injected into the UPLC-MS system for metabolomic analysis. Additionally, 10 μl from each sample was pooled to generate quality control (QC) samples for the UHPLC-MS/MS analysis.

### Instrumentation and Conditions for UHPLC-Q-TOF/MS Analysis

The separation was performed on an Agilent^®^1290 Infinity II (Agilent Technologies Inc., USA) using a Waters ACQUITY HSS T3 column (100 × 2.1 mm, i.d. 1.8 µm) maintained at 30°C. AB SCIEX^®^ TripleTOF 5600+ Plus ultra-performance liquid chromatography-tandem mass spectrometer (UHPLC-Q-TOF/MS) was used to acquire the MS/MS spectra on information-dependent basis during the LC/MS experiment. In this mode, acquisition software (Analyst TF1.7 software) continuously evaluates the full scan survey MS data as it collects and triggers the acquisition of MS/MS spectra depending on preselected criteria (20). The mobile phase containing 0.1% formic acid was composed of water (A) and acetonitrile (B) at a flow rate of 0.3 ml/min. A gradient program was used as follows (time, min/A%): 0.5/99, 1.5/99, 7/1, 15/1, 15.5/99, 20/99. The injection volume was 5 μl in partial loop mode. Electrospray ionization mass spectrometry (ESI-MS) was operated in negative/positive ion mode under the following operating parameters: curtain gas, 35; ion source gas 1, 55; ion source gas 2, 55; temperature, 550; declustering potential, ± 80; collision energy, ± 40; accumulation time, 0.16 s. The pooled QC represented the sample matrix and metabolite composition of the samples, which were used to construct the calibration curves and to judge precision, stability and recovery are within the acceptable range.

### Analysis of Differential Metabolites and Metabolic Pathways

The analysis workflow of differential metabolites and metabolic pathways included five main steps: data acquisition, spectral processing, metabolite identification, analysis of metabolic pathways and diagnostic potential of differential metabolites (21, 22). The UHPLC-Q-TOF/MS method was used for data acquisition in all samples in the positive and negative ion modes, and the processed data were subjected to multivariate statistical analysis. Initially, individual peaks were filtered to remove noise based on the relative standard deviation or coefficient of variation. Then, the missing values were replaced with half of the corresponding minimum values. Additionally, the IS normalization method was used for data analysis. The analytical platform used for metabolomic data analysis was provided by Biotree Biotech Co., Ltd. (SIMCA15.0.2 software package) and Dashuo Biotech Co., Ltd. (ONE-MAP). Linear transformation was used to preserve the variance of the original data in the lower dimensionality of the output data using principal component analysis (PCA) score plots, and the outliers were identified by Hotelling’s T-squared distribution (23). Significantly differential metabolites were identified using partial least squares-discriminant analysis (PLS-DA), and 200 random permutation tests were carried out to avoid overfitting of the PLS-DA models (24). The metabolites were considered significantly altered based on the results of PLS-DA and heat map based on the *t*-test threshold (*p* < 0.05), fold-change threshold (FC >1.5 or FC< 2/3), and variable importance in the projection (VIP > 1). Exact molecular weights of the metabolites (molecular weight error < 20 ppm) were confirmed, and they were matched and annotated in the standard database, custom databases (Metlin, MassBank, LipidMaps, Mzclound, and HMDB databases), and other integrated databases to obtain accurate metabolite information. The metabolic pathways possibly associated with GC were identified by searches for pathways of metabolites in commercial databases, including KEGG and MetaboAnalyst (25). Finally, the diagnostic performance of differential metabolites was analyzed by receiver operating characteristic (ROC) curve and PLS-DA.

### Fasting Lipid Profile Assays

The serum fasting lipid profile was determined at diagnosis during routine preoperative examination. Blood was collected into EDTA-coated tubes, and the serum levels of total cholesterol (TC), triglycerides (TG), low-density lipoprotein cholesterol (LDL-C), high-density lipoprotein cholesterol (HDL-C), apolipoprotein A1 (ApoA1), and apolipoprotein B (ApoB) were measured automatically by a Roche Cobas 8000 modular analyzer.

### Statistical Analysis

All data were statistically analyzed using SPSS 25.0 software (International Business Machines Corp., USA). Data with a normal distribution are expressed as the mean ± standard deviation. Multiple groups with equal variances were compared by one-way ANOVA followed by LSD *t*-test; data with unequal variances were compared by Welch’s approximate analysis of variance followed by Dunnett’s T3 test. Bonferroni–Holm method was used to counteract the problem of multiple comparisons. A *P* value <0.05 indicated that the difference is statistically significant.

## Results

### Multivariate Analysis of Metabolomic Data of Serum Samples of the Control, CG and GC Groups

**Figure 1:**
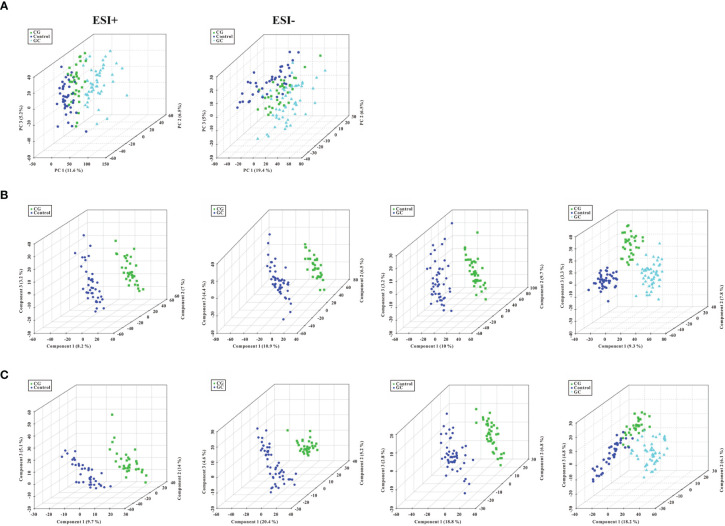
Score plots of PCA model and the first three latent components of PLS- DA model. **(A)** Three-dimensional score plot of the PCA model of all samples in the ESI+ and ESI− ion modes. **(B, C)** Score plots for the first three latent components of the PLS-DA model for CG *vs* Control, CG *vs* GC, GC *vs* Control, and CG plus GC *vs* Control in the ESI+ and ESI− ion modes, respectively. Healthy volunteers, control, CG, chronic gastritis; GC, gastric cancer.

**Figure 2:**
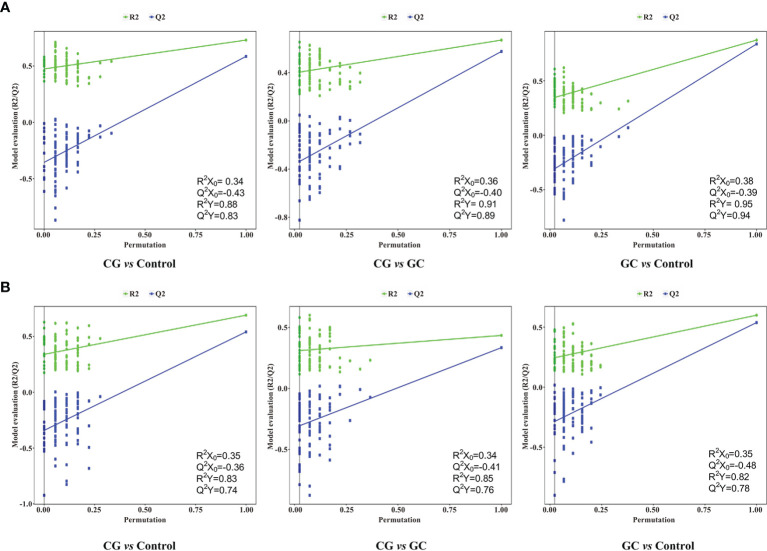
Permutation tests of the PLS-DA model for CG *vs* Control, CG *vs* GC, and GC *vs* Control in the **(A)** ESI+ and **(B)** ESI− ion modes. Random permutations (a total of 200) were used to evaluate whether a possibility of overfitting in the PLS-DA model. The statistical parameters R^2^X_0_, Q^2^X_0_, R^2^Y, and Q^2^Y were used for the analysis of the multivariate models.

Metabolic profiling performed in the present study included sample preparation, metabolite extraction, and LC/MS analysis. A total of 123 serum samples and 15 QC samples were used for metabolomic analysis by UHPLC-Q-TOF/MS. Representative total ion chromatograms (TIC) in positive and negative ion modes were shown in **Supplementary Figures S1A, B**, respectively. The response of clenbuterol in the positive ion mode and chloramphenicol in the negative ion mode used as IS were shown in **Supplementary Figures S1C, D**, respectively. The score plot of the PCA model was used for the first three principal component analyses of the data of the control, CG, and GC groups in the positive (ESI+) and negative (ESI−) modes (**Figure 1A**). The results of the PCA score plot indicated that the principal components were effectively separated. Furthermore, the PCA score plot of the pooled QC samples (**Supplementary Figures S2A, B**) and the relative standard deviation of IS (**Supplementary Figures S2C, D**) in the ESI+ and ESI− modes indicated that the analysis had satisfactory stability and repeatability of metabolomic datasets. PLS-DA with a better discriminative power than that of PCA was performed to characterize the metabolic profiles based on class information. All significant ions in the ESI+ and ESI− modes were merged and imported into the SIMCA15.0.2 software package and ONE-MAP. Comparison of all group pairs using the score plots for the top three latent components of the PLS-DA model showed significant clustering, which demonstrated a clear separation in the ESI+ (**Figure 1B**) and ESI− (**Figure 1C**) modes. In particular, the first three latent components of PLS-DA model in the control, CG, and GC groups were mainly distributed from left to right similar to a trend in the changes in the main principal components and were associated with disease progression. To avoid overfitting effect of PLS-DA models, 200 random permutation tests were performed; the results indicated the absence of overfitting with R^2^X_0_ (0.33–0.38), Q^2^X_0_ (−0.36 to −0.43), R^2^Y (0.82–0.95), and Q^2^Y (0.74–0.94), demonstrating the differences in metabolic profiles between the control, CG, and GC groups (**Figure 2**). These results confirmed high goodness of fit and predictive capability of the PLS-DA models. Therefore, two models, PCA and PLS-DA were sufficient to characterize the results of serum metabolite profiling.

### Analysis of Differential Metabolites and Pathways in the Control, CG, and GC Groups

**Figure 3:**
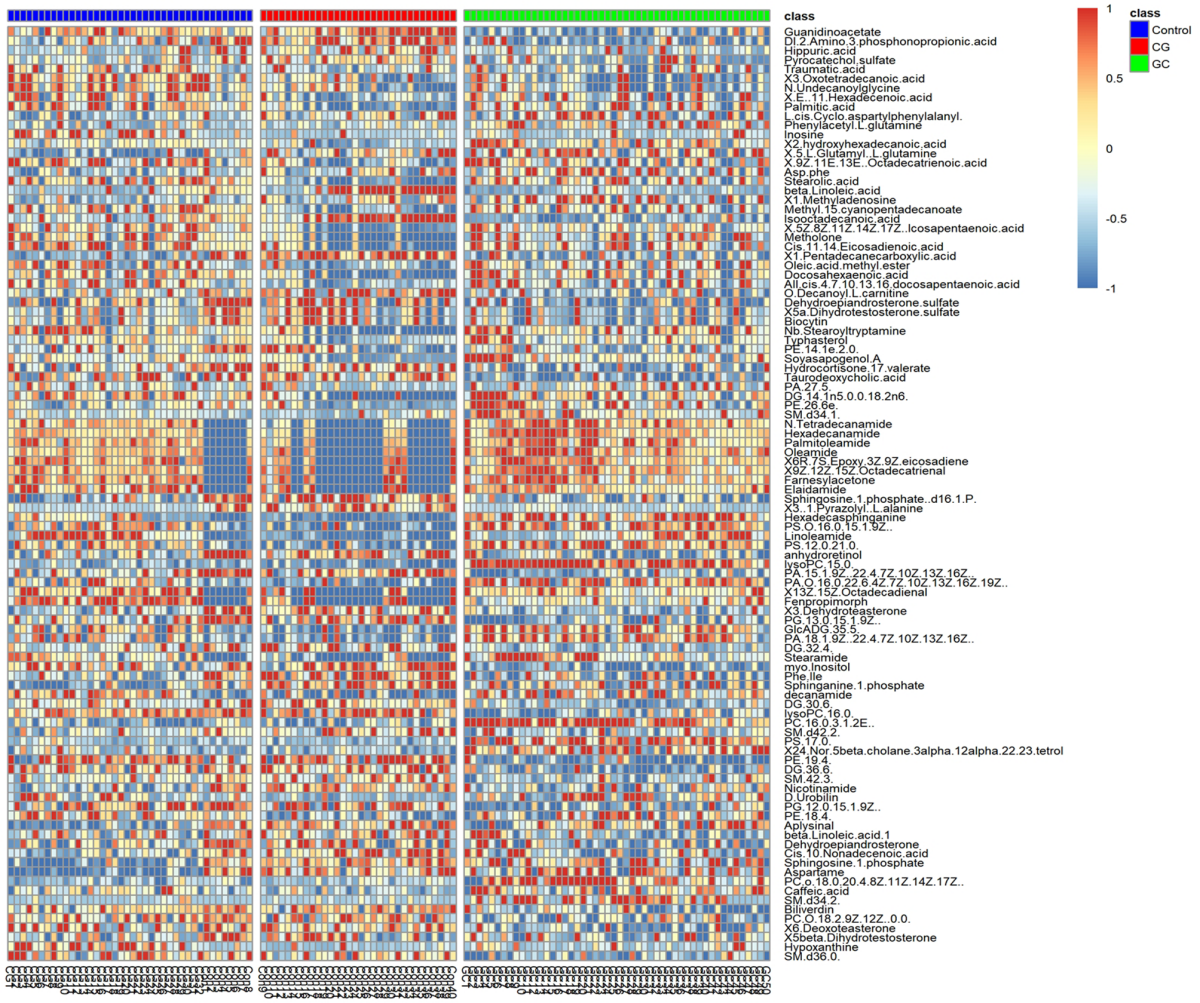
Heat map of the 100 significantly differential metabolites in the serum in the control (purple), CG (red), and GC (green) groups. Metabolites were included based on VIP > 1, FC > 1.5 or FC < 2/3, and *p* < 0.05. The colors from blue to red indicate the relative contents of the metabolites in the three groups.

Peaks were aligned, and the missing values were removed (26) to identify a total of 7,445 peaks in the ESI+ mode and 2,745 peaks in the ESI− mode based on the MS/MS data. Then, qualitative identification was performed using three strategies, namely, standard compounds databases, publicly available databases, and several integrated databases for molecular structure/fingerprint prediction. A total of 1,884 metabolites in ESI+ mode and 556 metabolites in ESI− mode were identified and subjected to statistical analysis. A total of 100 differentially accumulated metabolites were identified based on variable importance in the projection (VIP) >1 in the loading plot, FC >1.5 or FC <2/3, and *p* < 0.05. Global overview of metabolism features was shown in the heat map that included 100 differential metabolites in the three groups (**Figure 3**). Interestingly, lipids and lipid-like molecules were the most predominating metabolites including N-Hydroxy arachidonoyl amine, SQDG (29:3), hexadecasphinganine, hypoxanthine, 3-benzoyloxy-11-oxo-12-ursen-28-oic acid, MGDG (28:8), 2-methoxy-estradiol-17beta 3-glucuronide, PG [14:1(9Z)/14:1(9Z)], MGDG (20:2), traumatic acid, stearic acid, stigmastentriol, linoleamide, and other compounds. Then, we performed pathway enrichment and topological analysis based on 100 differential metabolites in serum. The number of differential metabolites matching the signal pathway (Hits), -ln(*P*) value, and pathway impact score (Impact) was used to demonstrate the enrichment of different metabolites mainly in sphingolipid metabolism, glycerophospholipid metabolism, arachidonic acid metabolism, tryptophan metabolism, steroid hormone biosynthesis, phenylalanine metabolism, linoleic acid metabolism, retinol metabolism pathways, and other pathways. Most of the significantly altered metabolic pathways were correlated with lipid metabolism, and detailed information about the pathways is shown in **Table 2**.

**Table 2:** Significantly altered metabolic pathways in the control, CG and GC groups.

Pathway name	KEGG.id	Hits^1^	-ln(*P*)	Impact^2^
Sphingolipid metabolism	hsa04071	5	7.75	0.28
Glycerophospholipid metabolism	hsa00564	5	5.40	0.26
Arachidonic acid metabolism	hsa00590	5	3.90	0.23
Tryptophan metabolism	hsa00380	4	1.67	0.16
Steroid hormone biosynthesis	hsa00140	4	1.39	0.11
Phenylalanine metabolism	hsa00360	3	4.55	0.28
Linoleic acid metabolism	hsa00591	2	2.90	0.66

^1^Hits, the number of differential metabolites matching the pathway; ^2^Impact, impact value of metabolic pathway determined by topology analysis.

### Identification of Metabolites as Candidate Biomarkers for CG and GC Diagnosis

**Figure 4:**
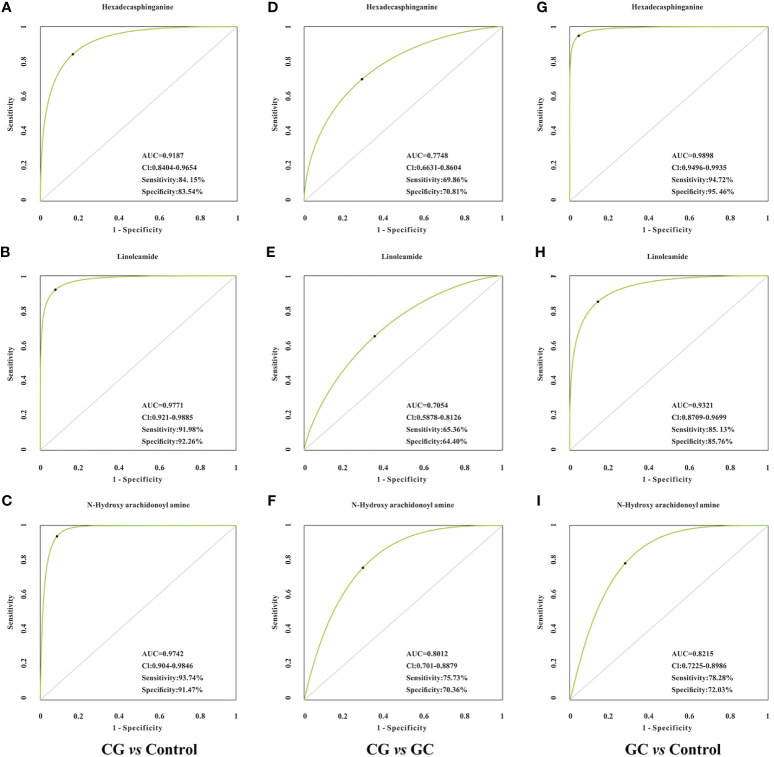
ROC curve analysis of the candidate biomarkers for CG or GC. Individual ROC curves and peak areas for hexadecasphinganine **(A, D, G)**, linoleamide **(B, E, H)** and N-Hydroxy arachidonoyl amine **(C, F, I)**. AUC (0.5−0.7), low accuracy; AUC (0.7−0.9), moderate accuracy; AUC (> 0.9), high accuracy. From the panel, hexadecasphinganine, linoleamide, and N-Hydroxy arachidonoyl amine displayed high efficiency for distinguishing patients with CG or GC from healthy control, but moderate efficiency when used to distinguish CG group from GC group.

**Figure 5:**
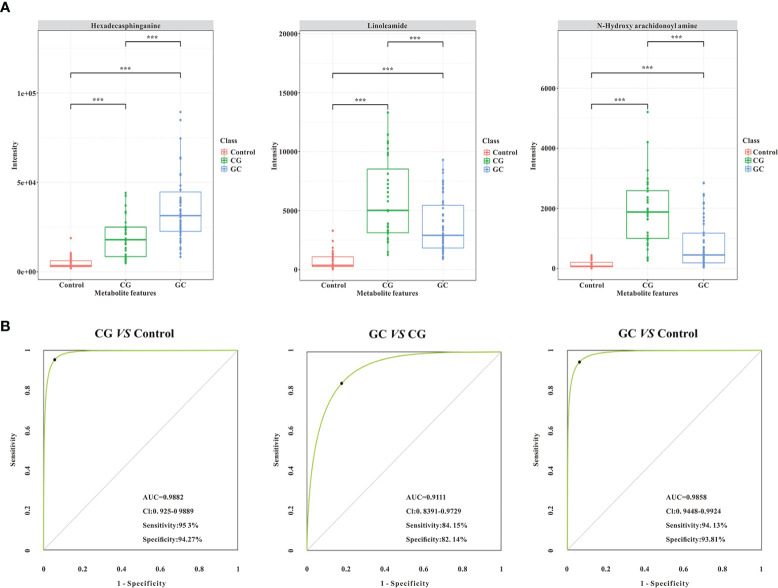
Validation of the combination of the three lipid metabolites (AUC > 0.90, ^***^p < 0.005) as potential biomarkers by ROC curve analysis. **(A)** The comparison of normalized intensity peak areas of three candidate biomarkers (hexadecasphinganine, linoleamide, and N-Hydroxy arachidonoyl amine) in the control (orange), CG (green), and GC (blue) groups. **(B)** Potential diagnostic performance of the three identified metabolites (AUC > 0.90, ^***^p < 0.005). The sensitivity and specificity values were optimized by PLS-DA. As shown in the panels, the area under the ROC curve of the combination of the three candidate biomarkers is significantly increased, suggesting that the combination of the three parameters has the highest diagnostic accuracy, especially for distinguishing GC patients from CG patients.

Lipid metabolites, including linoleamide, N-Hydroxy arachidonoyl amine, and hexadecasphinganine were significantly upregulated (FC > 1.5) in both CG and GC patients compared with those in healthy subjects. Comparison of the GC group with the CG group indicated that the serum levels of 3-benzoyloxy-11-oxo-12-ursen-28-oic acid, PG [14:1(9Z)/14:1(9Z)], 2-methoxy-estradiol-17beta 3-glucuronide, MGDG (20:2), SQDG (29:3), MGDG (28:8), and hexadecasphinganine were elevated; however, the serum level of N-Hydroxy arachidonoyl amine decreased. Then, we identified candidate biomarkers for the discrimination of CG or GC from healthy healthy subjects; the diagnostic potentials of 100 differential metabolites were tested by receiver operating characteristic curve (ROC) analysis. According to the basic principle for ROC analysis (27), differential metabolites with AUC >0.70 were chosen as candidate markers (**Table 3**). The data indicated that hexadecasphinganine (**Figures 4A, G**), linoleamide (**Figures 4B, H**), and N-Hydroxy arachidonoyl amine (**Figures 4C, I**) were able to highly efficiently discriminate CG or GC between CG or GC patients and healthy subjects (AUC > 0.90); discrimination of CG from GC by the three metabolites was characterized by moderate efficiency (AUC = 0.7047–0.8012, **Figures 4D–F**). So, sensitivity and specificity of the three markers were optimized according to the PLS-DA, and a combination of these markers improved sensitivity and specificity (AUC = 0.9882, CG *vs* Control; AUC = 0.9111, GC *vs* CG; AUC= 0.9858, GC *vs* Control) (**Figure 5B**). The peak areas of hexadecasphinganine, linoleamide, and N-Hydroxy arachidonoyl amine in the control, CG, and GC groups were significantly different (**Figure 5A**). These results suggested that hexadecasphinganine, linoleamide, and N-Hydroxy arachidonoyl amine can be used as candidate biomarkers for the diagnosis of CG or GC.

**Table 3:** Differential metabolites identified by *t*-test and ROC curve analysis in two groups.

Group	Compounds	FC^1^	log2(FC)	VIP	AUC	CI	*P*-value	Adjusted *P^2^*
**CG *vs* Control**	Linoleamide	8.63	3.11	3.02	0.9771	0.92-0.99	9.31E-10	1.75E-06
	N-Hydroxy arachidonoyl amine	13.75	3.78	3.10	0.9742	0.90-0.98	3.89E-10	7.29E-07
	Hexadecasphinganine	3.85	1.95	2.69	0.9187	0.84-0.97	6.24E-08	1.17E-04
	Hypoxanthine	4.70	2.23	2.10	0.8331	0.74-0.94	1.47E-05	8.76E-03
**GC *vs* CG**	N-Hydroxy arachidonoyl amine	0.43	-1.23	1.69	0.8012	0.70-0.89	1.57E-05	2.94E-02
	SQDG (29:3)	3.10	1.63	1.68	0.7867	0.69-0.87	3.74E-06	2.23E-03
	Hexadecasphinganine	1.83	0.87	1.53	0.7748	0.66-0.86	7.33E-06	1.37E-02
	3-Benzoyloxy-11-oxo-12-ursen-28-oic acid	3.74	1.90	1.69	0.7416	0.63-0.84	5.44E-06	1.02E-02
	MGDG (28:8)	2.09	1.07	1.59	0.7403	0.63-0.84	3.52E-05	2.10E-02
	2-Methoxy-estradiol-17beta 3-glucuronide	3.52	1.82	1.71	0.7396	0.63-0.84	3.13E-06	1.87E-03
	PG (14:1(9Z)/14:1(9Z))	3.58	1.84	1.67	0.7311	0.62-0.83	1.11E-05	6.62E-03
	MGDG (20:2)	3.11	1.64	1.69	0.7147	0.60-0.81	7.39E-06	4.40E-03
**GC *vs* Control**	Hexadecasphinganine	7.03	2.81	2.42	0.9898	0.95-0.99	1.19E-15	2.23E-12
	Linoleamide	5.35	2.42	2.16	0.9321	0.87-0.97	5.04E-12	9.44E-09
	Stigmastentriol	4.04	2.01	2.04	0.9015	0.82-0.96	1.61E-10	3.02E-07
	N-Hydroxy arachidonoyl amine	5.87	2.55	1.74	0.8215	0.72-0.90	6.80E-07	1.27E-03

^1^FC, FC > 1.5 indicates the upregulated serum level, FC < 2/3 indicates the downregulated serum level. ^2^Adjusted P, Adjusted P indicates the Bonferroni–Holm adjusted P values.

### Serum Level of Fasting Lipid Profile in Patients With CG or GC When Compared With Control

The fasting lipid profile was determined to verify the relationships between lipid metabolism and GC (**Table 4**). Interestingly, the levels of TC, HDL-C, LDL-C and Apo-A1 were statistically different between the groups (*p* < 0.05). Subsequent statistical analysis showed that the serum levels of TC, HDL-C and Apo-A1 in patients with GC were substantially lower than those of patients with CG indicating a possible association between decreased TC, HDL-C, and ApoA1 levels and the progression of CG to GC (*p* < 0.05). The serum concentrations of TC, HDL-C and Apo-A1 in the GC group were significantly decreased compared with those in CG group (*p* < 0.05). However, there were no statistically significant differences in the levels of TG and Apo-B between the three groups (*p* > 0.05). In general, decreased TC, HDL, and Apo-A1 levels may be associated with the progression of GC.

**Table 4:** Serum lipids and apolipoproteins profile for samples.

Group	Control (n = 40)	CG (n = 32)	GC (n = 51)	*p* value
TC (mmol/L)	4.66 ± 0.57	4.6 7± 0.86	3.98 ± 0.96^*#^	<0.001
TG (mmol/L)	1.1 8 ± 0.36	1.36 ± 0.58	1.39 ± 0.60	0.087
HDL-C (mmol/L)	1.42 ± 0.23	1.59 ± 0.43	1.16 ± 0.50^*#^	<0.001
LDL-C (mmol/L)	2.71 ± 0.50	2.59 ± 0.70	2.27 ± 0.80^*^	0.009
Apo-A1 (g/L)	1.54 ± 0.14	1.63 ± 0.30	1.16 ± 0.40^*#^	<0.001
Apo-B (g/L)	0.91 ± 0.12	0.90 ± 0.22	0.91 ± 0.26	0.983

Compared with the control group, ^*^p < 0.05. Compared with the CG group, ^#^p < 0.05.

## Discussion

Cancer cells frequently display fundamentally altered cellular metabolism, which provides the biochemical basis and directly contributes to tumorigenicity and malignancy. Therefore, cancer metabolism has recently become a subject of considerable interest for the pharmaceutical industry and clinical research. However, a systematic understanding of cancer metabolism remains a challenge. The primary goal of this study was to investigate the serum metabolic features and identify candidate biomarkers for gastric disease.

Most candidate biomarkers identified in the present study were lipid-related molecules (**Figure 3**). Most metabolic pathways were involved in lipid metabolism, including sphingolipid metabolism, glycerophospholipid metabolism, and arachidonic acid metabolism (**Table 2**). To further study the relationship of lipid metabolism with the development of GC, fasting lipid profile was prospectively assessed. The serum TC, HDL-C, and ApoA1 levels decreased in patients with GC compared with those in healthy subjects or CG patients (**Table 4**). The serum levels of these metabolites in patients with GC were considerably lower than those in patients with CG indicating a possible association between decreased TC, HDL-C, and ApoA1 levels with the progression of CG to GC. A decrease in the lipid profile in cancer patients may be due to increased utilization of lipids by neoplastic cells in membrane biogenesis, which is consistent with the findings of other studies (28, 29). The results of untargeted metabolomic analysis and fasting lipid profile indicated that lipid metabolism may be associated with the development of CG to GC. The development of gastric cancer is a multistep process, and CG is the initial step of the precancerous cascade (30, 31). However, how gastritis is initiated and transformed to GC remains unclear. Many types of cancer are caused by infection, chronic irritation, and inflammation (32). Therefore, it is important to understand how inflammation contributes to the physiological and pathological processes of cancer.

Many studies have investigated the relationships of lipid metabolism with inflammation and tumorigenesis (33, 34). The engagement of specialized pro-resolving lipid mediators (SPMs) has been reported to be involved in the inflammatory response; these lipid mediators and their signaling pathways are the key components of an important endogenous anti-inflammatory and immunoregulatory pathway that promotes the resolution of inflammation. Recently, a novel signaling pathway, ALOXs-GPR32-STAT3, was shown to control gastric cancer angiogenesis through the production of specialized SPMs (35). Arachidonic acid is the precursor of diverse inflammatory molecules (36), and stearic acid has been shown to be positively correlated with proinflammatory IL-8 (37). Moreover, lipid metabolites are important components of the human body that have biological and functional roles, such as sources of energy *via β*-oxidation and dominant components of the cellular membranes (38, 39). Because rapidly proliferating cancer cells can survive by enhancing exogenous lipid uptake and activating endogenous lipid synthesis to supply energy, upregulated levels of lipid metabolites were detected in the serum of CG or GC patients. In addition, lipid metabolism, such as sphingolipid metabolism, glycerophospholipid metabolism, and linoleic acid metabolism, can influence cancer metastasis, therapeutic effects, and prognosis (40–42).

Altered metabolism of lipids is currently considered a hallmark characteristic of many malignancies, and lipids have raised growing interest as potential biomarkers in many clinical conditions. Therefore, we investigated the serum diagnostic potentials of these significantly different metabolites. The results indicated that linoleamide and N-Hydroxy arachidonoyl amine can be used as candidate biomarkers for CG, and hexadecasphinganine and linoleamide can be used as candidate biomarkers for GC. The combination of these biomarkers can increase the diagnostic accuracy for the discrimination of CG *versus* GC. Thus, single biomarkers have high sensitivity and specificity for diagnosis of CG or GC *versus* healthy subjects. However, the results obtained using a combination of the three biomarkers enhanced the discrimination provided by diagnostic tests based on single markers for differential diagnosis of CG *versus* GC. This study is the first to report that three lipid compounds (hexadecasphinganine, linoleamide, and N-Hydroxy arachidonoyl amine) have high potential diagnostic value and may be used as candidate biomarkers for CG or GC diagnosis. However, the pathophysiological role of these three metabolites in the development of GC has not been reported, although these compounds have been investigated in other diseases. For example, linoleamide, a representative of fatty acids, has anti-inflammatory effects (43). Hexadecasphinganine, a potential biomarker for Alzheimer’s disease (44), plays major roles in sphingolipid signaling to regulate important cellular processes, including cell proliferation, metabolism, differentiation, and protein synthesis (45). However, the pathophysiological role of N-Hydroxy arachidonoyl amine has not been reported. Thus, some of the differential metabolites and metabolic pathways identified in our study were consistent with the data of previous studies with some limitations because of the differences among individual participants and the limited number of patients. In the follow-up study, we hope to verify the accuracy of the diagnostic values of our identified panels and explore the detailed molecular mechanism of lipid metabolism promoting the transition from CG to GC.

## Conclusions

This study provides new insights into the changes in serum metabolites during the development of gastric diseases. We demonstrated that the serum levels of metabolites in patients with chronic gastritis or gastric cancer were enriched in lipid metabolism and significantly different from those in the healthy subjects. The levels of lipid metabolism were different in the CG and GC groups, which enhanced the understanding of the pathogenesis of gastric disease. Additionally, differential lipid metabolites validated in this study may be as diagnostic biomarkers for the diagnosis and classification of this disease. Follow-up investigations are expected to define the diagnostic parameters of these candidate biomarkers *via* targeted metabolomic analysis of additional clinical samples.

## Data Availability Statement

Publicly available datasets were analyzed in this study. The original mass spectrometry data have been deposited to the ProteomeXchange Consortium (http://proteomecentral.proteomexchange.org) *via* the iProX partner repository (46) with the dataset identifier PXD023847.

## Ethics Statement

The studies involving human participants were reviewed and approved by the Ethics Committee of Mianyang Central Hospital. The patients/participants provided their written informed consent to participate in this study.

## Author Contributions

Conceptualization, LY. Methodology, LY. Software, QF. Validation, BX, YL, and QL. Writing-original draft preparation, LY. Writing—review and editing, BX, LY, and JF. Project administration, JF. Funding acquisition, LY. All authors contributed to the article and approved the submitted version.

## Funding

This study was funded by the China Postdoctoral Science Foundation (2019M653429), Sichuan Provincial Health and Family Planning Commission Research Project (20PJ257), and Incubation Project of Mianyang Central Hospital (2019FH09 and 2020XGZX020).

## Conflict of Interest

The authors declare that the research was conducted in the absence of any commercial or financial relationships that could be construed as a potential conflict of interest.

## Supplementary Material

Supplementary Figure 1 The established metabolomics approach for the analysis of the serum samples. **(A)**, **(B)** are the representative UHPLC-Q-TOF/MS total ion chromatograms of the serum samples in the ESI+ and ESI− ion modes, respectively. Clenbuterol and chloramphenicol were selected as internal standards (ISs) for the positive and negative ion modes, respectively. The responses of the serum samples to clenbuterol and chloramphenicol at the concentrations of 500 ng/ml are shown in **(C)**, **(D)**, respectively.

Supplementary Figure 2 The PCA model with QC and the relative standard deviation of IS for all samples. **(A)**, **(B)** are the PCA model for all samples with QC in the ESI+ and ESI− modes, respectively. **(C)**, **(D)** are the relative standard deviation of IS for all samples in the ESI+ and ESI− modes, respectively.

Supplementary Figure 3 The intensity of peak areas for hexadecasphinganine **(A, D and G)**, linoleamide **(B, E, H)** and N-Hydroxy arachidonoyl amine **(C, F, I)** when compared to each two groups, ****p* < 0.005.

## Abbreviations

CG, Chronic gastritis; GC, Gastric cancer; ESI, Electrospray ionization; UHPLC-Q-TOF/MS, Ultra-high-performance liquid chromatography–tandem quadrupole/time-of-flight mass spectrometry; PCA, Principal component analysis; PLS-DA, Partial least squares discriminant analysis; FC, Fold-change; VIP, Variable importance in the projection; IS, Internal standard; TC, Total cholesterol; TG, Triglycerides; HDL-C, High-density lipoprotein cholesterol; LDL-C, Low-density lipoprotein cholesterol; Apo A1, Apolipoprotein A1; Apo-B, Apolipoprotein B; ROC, Receiver operating characteristic; AUC, Area under curve; CI, Confidence interval; ALOXs, Activity of lipoxygenases; GPR32, G-protein coupled receptor 32; STAT3, Signal transducer and activator of transcription 3.
