# Supplementary figures and images for: Serum Metabolic Profiling Analysis of Chronic Gastritis and Gastric Cancer by Untargeted Metabolomics
Source: Front Oncol. 2021 Mar 11;11:636917. doi: 10.3389/fonc.2021.636917 (PMC7991914; doi:10.3389/fonc.2021.636917)

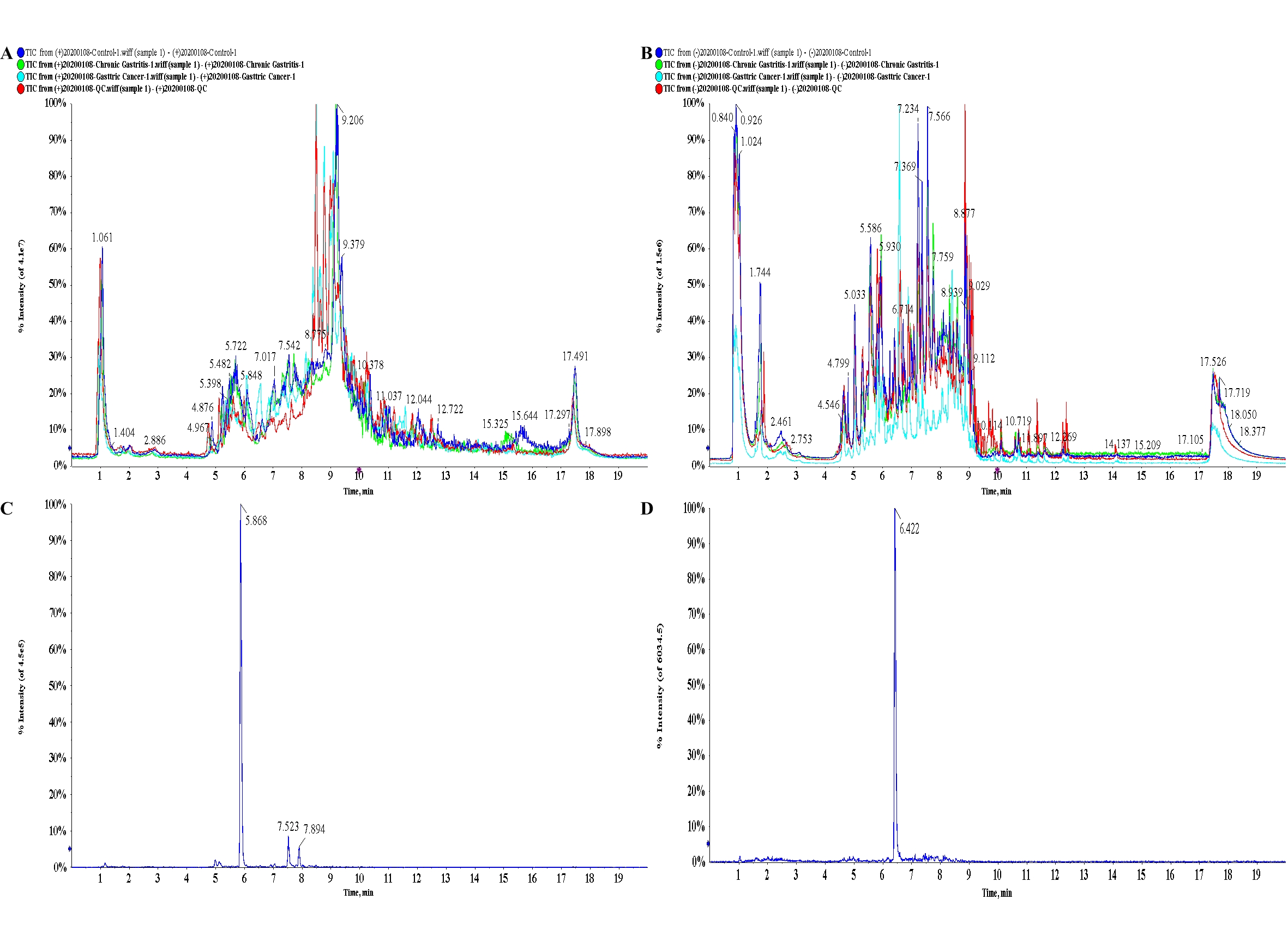

Supplement: Supplementary Figure 1 — The established metabolomics approach for the analysis of the serum samples. (A), (B) are the representative UHPLC-Q-TOF/MS total ion chromatograms of the serum samples in the ESI+ and ESI− ion modes, respectively. Clenbuterol and chloramphenicol were selected as internal standards (ISs) for the positive and negative ion modes, respectively. The responses of the serum samples to clenbuterol and chloramphenicol at the concentrations of 500 ng/ml are shown in (C), (D), respectively. [file Image_1.jpg]

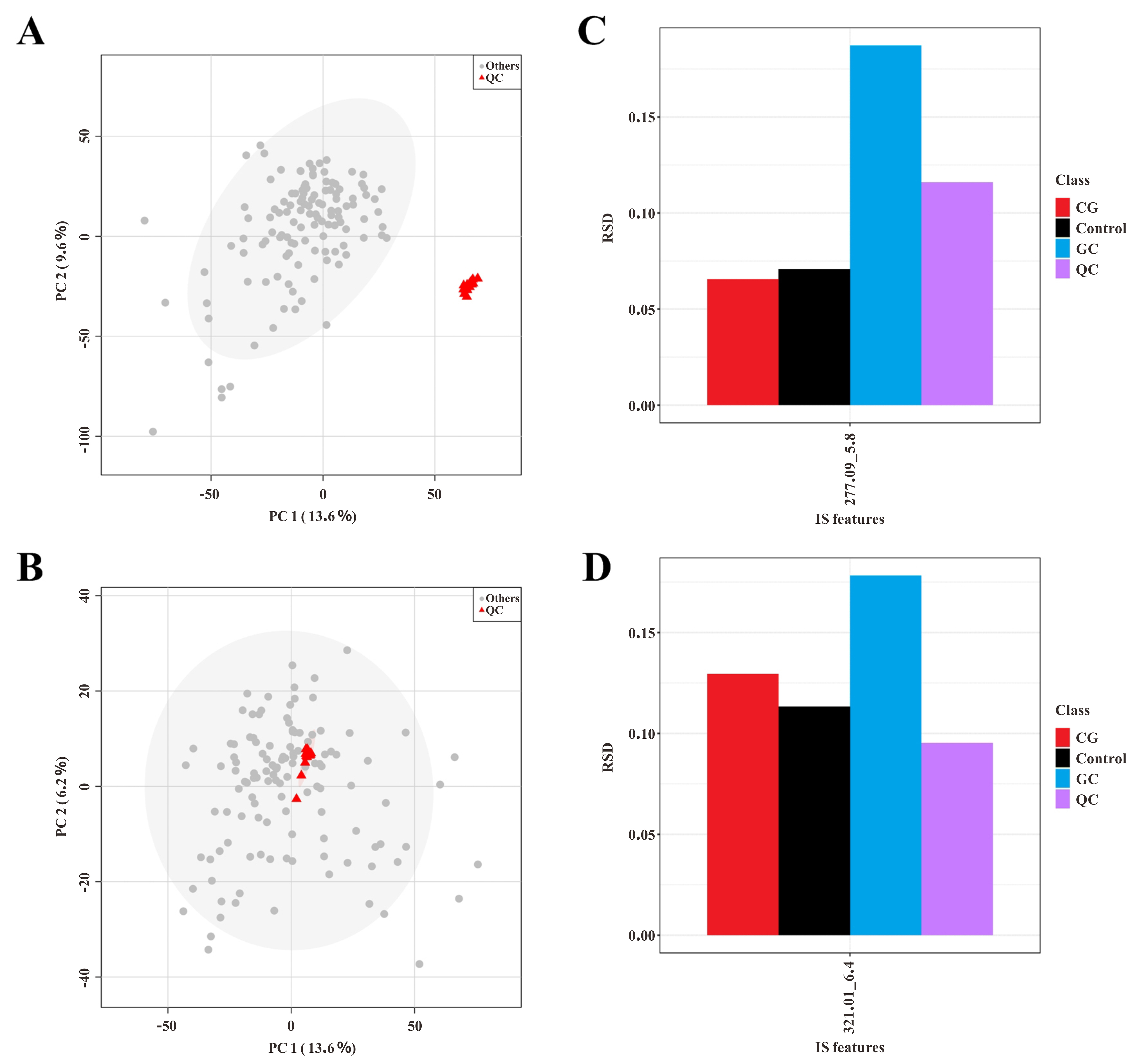

Supplement: Supplementary Figure 2 — The PCA model with QC and the relative standard deviation of IS for all samples. (A), (B) are the PCA model for all samples with QC in the ESI+ and ESI− modes, respectively. (C), (D) are the relative standard deviation of IS for all samples in the ESI+ and ESI− modes, respectively. [file Image_2.jpeg]

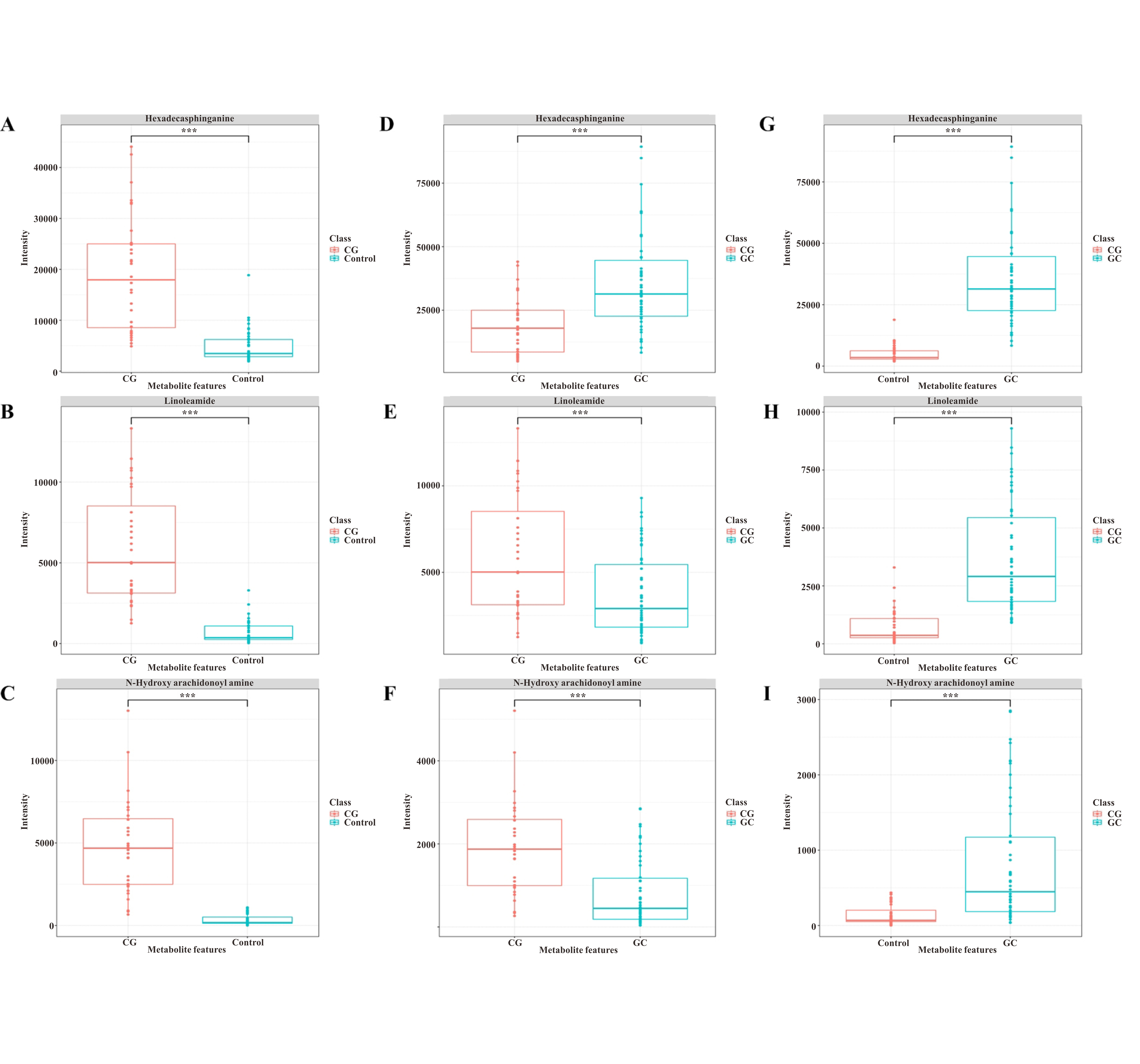

Supplement: Supplementary Figure 3 — The intensity of peak areas for hexadecasphinganine (A, D and G), linoleamide (B, E, H) and N-Hydroxy arachidonoyl amine (C, F, I) when compared to each two groups, ***p < 0.005. [file Image_3.jpeg]
